# Multisystem pathology in McLeod syndrome

**DOI:** 10.1111/neup.12935

**Published:** 2023-07-12

**Authors:** Katherine R. Schon, Dominic G. O’Donovan, Mayen Briggs, James B. Rowe, Lokesh Wijesekera, Patrick F. Chinnery, Jelle van den Ameele

**Affiliations:** 1 Department of Clinical Neurosciences, School of Clinical Medicine, University of Cambridge, Cambridge, UK; 2 Medical Research Council Mitochondrial Biology Unit, University of Cambridge, Cambridge, UK; 3 East Anglian Medical Genetics Service, Cambridge University Hospitals NHS Foundation Trust, Cambridge, UK; 4 Department of Pathology, Cambridge University Hospitals NHS Foundation Trust, Cambridge, UK; 5 Department of Clinical Neurophysiology, Cambridge University Hospitals NHS Foundation Trust, Cambridge, UK

**Keywords:** autopsy, McLeod syndrome, raised CK, *XK* gene

## Abstract

We present a comprehensive characterization of clinical, neuropathological, and multisystem features of a man with genetically confirmed McLeod neuroacanthocytosis syndrome, including video and autopsy findings. A 61-year-old man presented with a movement disorder and behavioral change. Examination showed dystonic choreiform movements in all four limbs, reduced deep-tendon reflexes, and wide-based gait. He had oromandibular dyskinesia causing severe dysphagia. Elevated serum creatinine kinase (CK) was first noted in his thirties, but investigations, including muscle biopsy at that time, were inconclusive. Brain magnetic resonance imaging showed white matter volume loss, atrophic basal ganglia, and chronic small vessel ischemia. Despite raised CK, electromyography did not show myopathic changes. Exome gene panel testing was negative, but targeted genetic analysis revealed a hemizygous pathogenic variant in the *XK* gene c.895C > T p.(Gln299Ter), consistent with a diagnosis of McLeod syndrome. The patient died of sepsis, and autopsy showed astrocytic gliosis and atrophy of the basal ganglia, diffuse iron deposition in the putamen, and mild Alzheimer’s pathology. Muscle pathology was indicative of mild chronic neurogenic atrophy without overt myopathic features. He had non-specific cardiomyopathy and splenomegaly. McLeod syndrome is an ultra-rare neurodegenerative disorder caused by X-linked recessive mutations in the *XK* gene. Diagnosis has management implications since patients are at risk of severe transfusion reactions and cardiac complications. When a clinical diagnosis is suspected, candidate genes should be interrogated rather than solely relying on exome panels.

## Introduction

McLeod syndrome is an ultra-rare neurodegenerative, multisystem disorder caused by X-linked recessive mutations in the *XK* gene.^1^ Around 250 cases are reported worldwide.^2^ It affects the central nervous system, peripheral nerves, skeletal muscle, heart, and red blood cells.^3^ The disorder was first described due to the distinct blood group phenotype in the Kell blood group system.^4^ A distinctive feature that may lead to early diagnosis is raised muscle creatinine kinase (CK),^5^ although neuromuscular features, including myopathy and axonal neuropathy, are often mild.^6^ The clinical presentation often resembles Huntington’s disease with progressive cognitive, behavioral, or psychiatric symptoms and a choreiform movement disorder.^3,7^ Seizures are the presenting feature in approximately 20% of cases,^6^ and the diagnosis is sometimes made incidentally upon blood group phenotyping.^8^ Neuromuscular symptoms may become more severe over time^9^ and can be a presenting feature, for example, severe weakness and muscle atrophy,^10^ persistent fatigue, excessive sweating or raised CK with unusual muscle biopsy findings,^11^ and rhabdomyolysis.^12^

The XK protein forms a heterodimer with the Kell glycoprotein in red blood cells.^3^ In other tissues such as the brain, heart, and skeletal muscle, Kell and XK are expressed independently from one another,^13^ and XK forms a complex with chorein/VPS13A,^14^ the protein implicated in autosomal recessive chorea-acanthocytosis.^7^ The latter condition has recently been renamed “VPS13A disease” to avoid confusion with McLeod syndrome and other inherited neurodegenerative disorders that can present with chorea and acanthocytosis.^15^ The pathogenesis of McLeod syndrome remains incompletely understood, and brain pathology findings have only been reported in four other cases.^16–21^ Comprehensive autopsy findings from multiple tissues have not been reported previously. We present the case of a man with genetically confirmed McLeod syndrome, including videos and autopsy findings from multiple tissues, including brain, skeletal, and cardiac muscle.

## Clinical Summary

A man presented to neurology in his thirties with elevated CK (between 2000 and 3000 μ/L), found incidentally when he underwent routine blood tests for alopecia. A thorough diagnostic work-up at that time, including muscle biopsy, did not provide a conclusive diagnosis. He was stable over the next 10 years and then stopped attending follow-up appointments. He developed generalized chorea, starting in his early fifties. He was hospitalized at age 60 with pneumonia due to dysphagia and was noted as having cognitive deterioration and behavioral changes. Collateral history revealed a lack of self-care, poor personal hygiene, difficulty managing finances, and lack of awareness of his surroundings (e.g., when crossing roads). There was no family history of movement disorder or neuromuscular disease. His father had been diagnosed with Lewy body dementia in his eighties, and two paternal uncles also suffered from dementia.

On examination in the neurology clinic at age 61, the patient had subtle dystonic choreiform movements in all four limbs and hands (Video S1) and oromandibular dyskinesia leading to dysphagia. He had reduced deep-tendon reflexes (Video S2), reduced light touch, temperature and vibration sense in the lower legs in a stocking pattern up to his knees, and an unusual broad-based gait (Video S3). On neuropsychological testing, he performed well in memory and visuospatial skills tests, but verbal initiation, social cognition, emotional recognition, and semantic fluency were impaired (Table S1).

**Fig 1:**
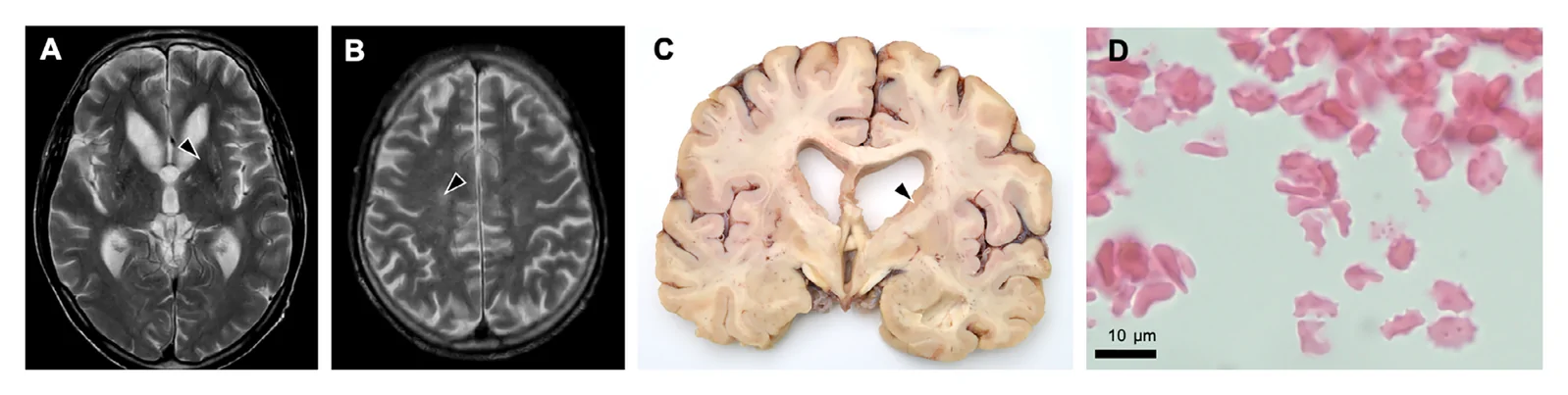
Brain MRI and autopsy findings. (A) T2-weighted MRI brain at age 61 showing chronic white matter volume loss and atrophic basal ganglia (arrowhead). (B) T2-weighted MRI brain at age 61 showing chronic small vessel ischemia (arrowhead). (C) Coronal section of the brain showing enlarged lateral and third ventricles and marked concavity of the head of the caudate nucleus (arrowhead). (D) Hematoxylin and eosin staining (×630, oil) showing acanthocytes in the veins of the choroid plexus of the lateral ventricle.

At age 61, CK was 1050 μ/L (RR40–320); CK-MM iso-enzyme 96% (RR97–100), full blood count, blood films (×2), liver function tests, creatinine, electrolytes, thyroid function tests, HbA1c and ANA, serum and urine copper, and caeruloplasmin levels were all normal. Brain magnetic resonance imaging (MRI) showed chronic white matter volume loss, atrophic basal ganglia (Fig. 1A), and chronic small vessel ischemia (Fig. 1B). Echocardiography showed moderate left ventricular dilatation. Nerve conduction studies were consistent with mild sensorimotor axonal neuropathy, and an electromyogram showed mild distal neurogenic changes (Table S2). Immuno-hematological analysis for the McLeod blood group phenotype was not performed.

Initial genetic testing focused on dominant disorders due to the strong family history of dementia. Genetic testing for Huntington’s disease, dentatorubral-pallidoluysian atrophy, common spinocerebellar ataxias, *JPH3, C9orf72*, and *POLG* did not reveal a diagnosis. Mitochondrial DNA extracted from muscle showed no depletion, sequence variants, or large-scale rearrangements. Further genetic testing found a hemizygous pathogenic variant in the *XK* gene (NM_021083.2): c.895C > T p.(Gln299Ter), which was previously reported in a large family with McLeod syndrome.^22^

Severe oromandibular dyskinesia caused 20 kg weight loss over the following year. He did not have typical tongue protrusion dystonia on examination. He had a percutaneous endoscopic gastrostomy inserted and moved to a specialist dementia home due to progressive behavioral and cognitive impairment. He passed away at age 63 due to MRSA sepsis.

## Autopsy Findings

### Neuropathology

At autopsy, the brain weighed 1400 g (RR for 61-year-old male 1240–1550^23^). Macroscopically, the cerebral hemispheres, cerebellum, and brainstem had a normal appearance. Coronal sections through the cerebrum showed moderate dilatation of the lateral and third ventricles and marked concavity of the head of the caudate nucleus resembling Huntington’s disease Vonsattel grade 4 (Fig. 1C). Acanthocytes were observed in large numbers in the lateral ventricle choroid plexus (Fig. 1D), but despite thorough inspection, these were not present in any other blood vessels examined.

**Fig 2:**
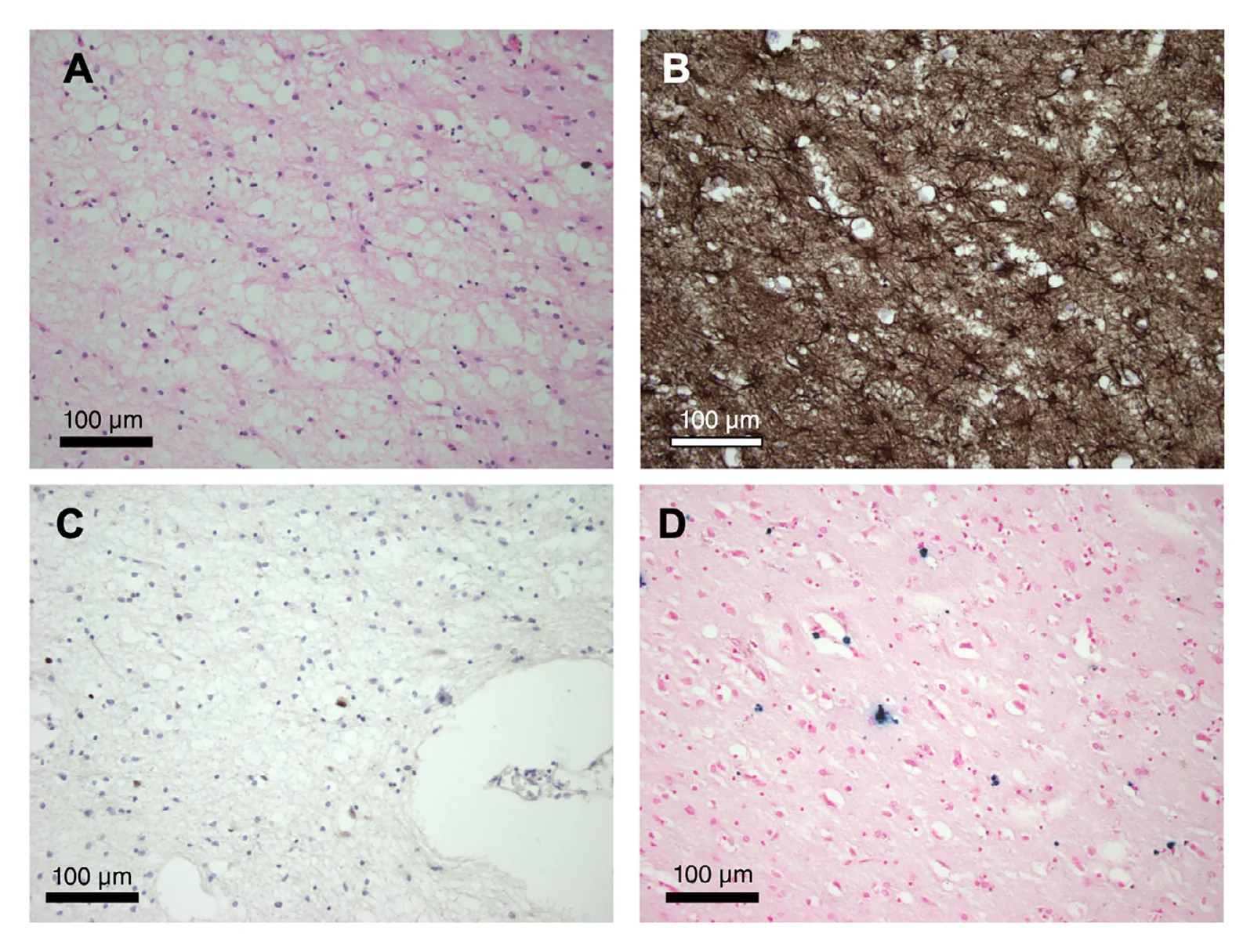
Brain histology. (A) Hematoxylin and eosin staining (×200) of caudate nucleus showing gliosis and severe neuronal loss. (B) Glial fibrillary acidic protein immunohistochemistry (×200) highlighting astrogliosis in the caudate nucleus. (C) NeuN ×200 stain of caudate nucleus showing that only approximately 10% of neurons remained. (D) Perls Prussian Blue (×200) showing mineral deposits in the caudate nucleus consistent with hemosiderin deposition.

Microscopic analysis of the brain confirmed marked atrophy of the caudate with astrogliosis, neuronal loss, and associated vacuolation of the neuropil (Fig. 2A–C). Gliosis and neuronal loss were also observed in the putamen and globus pallidus. Mild iron accumulation was noted in the caudate (Fig. 2D) and globus pallidus. There were signs of Alzheimer’s type pathology, with a moderate to high density of amyloid plaques within the striate, peri-striate cortex, inferior frontal, and frontoparietal region. Immunohistochemistry showed tau pathology in the form of neurofibrillary tangles and neuritic plaques within the superficial and deep cortical layers of the inferior frontal lobe and parietal lobe (Braak stage 4).

### Muscle pathology

**Fig 3:**
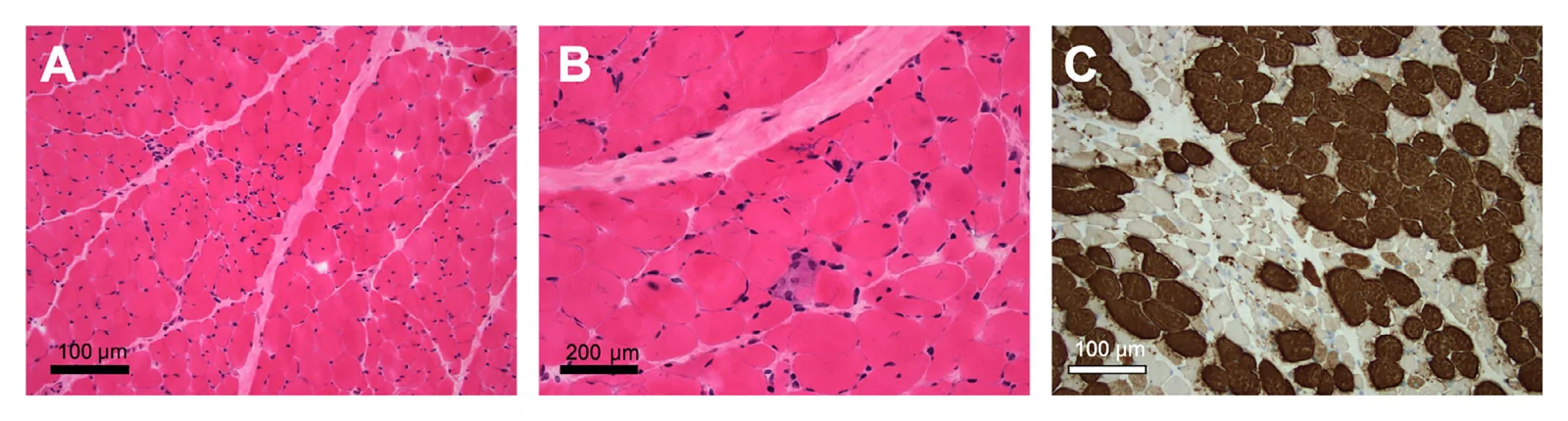
Muscle histology. (A) Hematoxylin and eosin (HE) staining (×200) of quadriceps muscle showing features of denervation and reinnervation with atrophy but no myonecrosis. (B) HE staining (×400) of pectoralis major muscle showing a basophilic fiber. (C) Slow myosin (×200) of quadriceps muscle showing the grouping of both slow and fast twitch muscle fibers indicative of chronic denervation.

Generalized muscle wasting was observed. Microscopically, there was fiber size variation with numerous angulated fibers, fiber type grouping (Fig. 3A–C), and type II fiber atrophy. Several fibers contained internalized nuclei (>3%). There were basophilic regenerating fibers (Fig. 3B), nuclear clusters, and rimmed vacuoles, and there was an increase in endomysial adipocytes. Muscle pathology was indicative of chronic neurogenic atrophy with non-specific myopathic changes.

### Multisystem features

The heart was enlarged (560 g; RR for 65 kg male 280–390^23^) due to concentric left ventricular hypertrophy with the compact myocardium of the left ventricular free wall measuring 1.5 cm. Microscopically, there was non-specific cardiomyopathy with fibrosis. The liver was enlarged (2025 g; RR1096–1930^23^) with a nutmeg appearance on slicing, consistent with passive venous congestion due to cardiac failure. The spleen was also enlarged (470 g; RR 97–320^23^). Other tissues examined were unremarkable. The cause of death was chronic congestive cardiac failure due to non-specific cardiomyopathy with superimposed hypostatic pneumonia related to MRSA septicemia.

## Discussion

The autopsy findings in this case report show a clear multisystem involvement. There was a discrepancy between the chronic, highly elevated CK, suggesting severe skeletal myopathy (with cardiomyopathy) but mostly chronic neurogenic changes in muscle histology. Elevated CK can be present in neurogenic muscle atrophy, including in amyotrophic lateral sclerosis^24^ and spinal muscular atrophy;^25^ however, the levels are rarely >1000 μ/L. Our findings are consistent with previous reports of muscle biopsy findings in 10 McLeod syndrome patients, which found variably severe neurogenic changes, such as fiber type grouping, and only 3/10 had a clearly increased number of internalized nuclei of >15%.^9^ Most patients have acanthocytosis on peripheral blood film.^7,9^ The method of preparation of the peripheral blood smear affects the level of acanthocytes observed. Higher levels of acanthocytes have previously been observed in healthy volunteers and people with movement disorders when isotonic dilution or wet preparations have been used.^26^ These methods may not have been used in our patient, in whom no acanthocytes were seen on peripheral blood films. At autopsy, acanthocytes were only observed in the vessels of the choroid plexus of the lateral ventricle.

Brain examination confirms previous findings of neuronal loss and astrocytic gliosis in the basal ganglia, with the caudate nucleus most severely affected.^16–19^ Mild Alzheimer’s type changes^17^ and iron deposits in the caudate nucleus^16^ have been reported previously. The significance of these changes is unclear, but these recurrent findings suggest that further study is warranted.

Early diagnosis of McLeod syndrome is important because the red blood cell antigen profile puts patients at risk of severe transfusion reactions.^3^ Cardiac complications, including dilated cardiomyopathy and tachyarrhythmias, are common and require screening and follow-up.^2,27^ Our case and others^5^ show that isolated hyperCKemia can be the first presentation of McLeod syndrome. When he was investigated for isolated hyperCKemia in the 1990s, no diagnosis was found. The diagnosis is likely to be made earlier in today’s era of next-generation sequencing,^28^ but we note that the *XK* gene is not currently present in commonly requested neuromuscular panels in the UK, including the UK Genomic Medicine Service Neuromuscular Disorders Panel (R381)^29^ or commercially available neuromuscular panels through Invitae or GeneDx. This illustrates that ultra-rare disorders may not be included in virtual panels, and different bioinformatic approaches (e.g., Exomiser)^30^ that do not rely on virtual gene panels can help diagnose ultra-rare conditions. In our case, a re-analysis of clinical exome data was requested once characteristic clinical features were recognized by an experienced clinician.

## Supplementary Material

Supplementary Table 1

Supplementary Table 2

Supplementary Video

Supporting Information

## Data Availability

The data that supports the findings of this study are available in the supplementary material of this article
