# Supplementary material for: Multisystem pathology in McLeod syndrome
Source: Neuropathology. Author manuscript; Available in PMC 2026 Oct 16. (PMC7619572; doi:10.1111/neup.12935)
Supplement: Supplementary file 1 [file EMS220163-supplement-Supplementary_Table_1.pdf]

Supplementary Table 1
Results of nerve conduction studies (NCS) and electromyography (EMG)

Sensory NCS

Nerve / Sites	Onset lat ms	Peak lat ms	Amplitude µV	Velocity m/s
**R Median and ulnar rec wrist**				
F3 Median	4.5	5.2	10.6	31.3
F5 Ulnar	2.8	3.1	2.1	43.5
Palm Median	2.6	3.5	20.2	30.7
**R Radial - (anti) rec thumb**				
Forearm	1.7	2.7	11.8	52.4
**L Radial - (anti) rec thumb**				
Forearm	2.1	2.8	10.6	48.0
**R Sural - rec Lat Malleol**				
Sural	2.9	3.9	5.9	34.9
**R Superficial peroneal - rec dorsum foot**				
Lateral Leg	No recordable potential			

Motor NCS

Nerve / Sites	Latency ms	Amplitude mV	Velocity m/s
**R Median - APB**			
Wrist	5.9*	8.9	
Elbow	12.0	8.6	45.9
**R Ulnar – ADM**			
Wrist	4.0*	9.5	
B.Elbow	9.2	8.3	45.6
A.Elbow	11.6	8.6	46.9
**R Peroneal – EDB**			
Ankle	10.0*	0.5	
Fib Head	20.8	0.5	31.4
Knee	24.0	0.0	32.0
**R Tibial – AH**			
Ankle	6.0*	4.5	
Knee	19.0	2.8	34.7

*Cold peripheries likely to account for mild distal conduction slowing

F Wave

Nerve	Min F Lat ms
R Median - APB	34.7
R Ulnar - ADM	35.0
R Peroneal - EDB	72.4
R Tibial - AH	59.3

Needle EMG

	Spontaneous		MUAP			Recruitment	Interference
Muscle	Fibs/PSWs	Other	Amp	Dur.	Polyphasia	Pattern	Pattern
R. Tibialis anterior	None	None	1+	1+	N	Reduced	Incomplete
R. Rectus femoris	None	None	1+	1+	1+	Reduced	Full, norm amp
R. First dorsal interosseous	None	None	1+	1+	1+	N	Full, norm amp
R. Biceps brachii	None	None	1+	1+	1+	N	Full, norm amp
