# Supplementary material for: Multisystem pathology in McLeod syndrome
Source: Neuropathology. Author manuscript; Available in PMC 2026 Oct 16. (PMC7619572; doi:10.1111/neup.12935)
Supplement: Supplementary file 2 [file EMS220163-supplement-Supplementary_Table_2.pdf]

Supplementary Table 2
Neuropsychological score sheet

In relation to estimated premorbid cognitive functioning

*	=	Mild Impairment
* *	=	Moderate Impairment
* * *	=	Marked Impairment

Test	Raw Score	Statistic Scores & Comments
**Verbal Memory**		
Doors & People – People		
T1 – T3 (Learning)	30/36	Scaled Score = 12
Delayed Recall	12/12	Scaled Score = 12
Doors & People – Names		
Recognition Test A	10/12	Scaled Score = 11
Recognition Test B	8/12	Scaled Score = 11
**Nonverbal Memory**		
Doors & People – Shapes		
T1 – T3 (Learning)	30/36	Scaled Score = 9
Delayed Recall	12/12	Scaled Score = 11
Doors & People – Doors		
Recognition Test A	11/12	Scaled Score = 10
Recognition Test B	8/12	Scaled Score = 11
**Verbal Skills**		
Test of Premorbid Functioning	45/70	Predicted premorbid 'FSIQ' = 98
Letter Fluency – FAS	28	Percentile = 30th
Semantic Fluency – Fruit & Vegetables	11	Scaled Score = 3 * *
**MLSE (v4.2) Naming**	3/6	Motor speech errors. Naming intact.
MLSE (v4.2) Single Syllable Repetition Polysyllabic Word Repetition Repeated Polysyllabic Word	4/4	Within normal limits
MLSE (v4.2) Repeat & Point		
Repetition Score	3/3	Within normal limits
Comprehension Score	3/3	Started singing about flowers
MLSE (v4.2) Non-Word Repetition	1/3	Motor speech errors.
MLSE (v4.2) Semantic Association	4/4	Within normal limits
MLSE (v4.2) Semantic Comprehension 1 (verbal)	5/5	Within normal limits
MLSE (v4.2) Semantic Comprehension 2 (visual)	2/4	Impaired
MLSE (v4.2) Irregular Word Reading	4/5	Motor speech error

MLSE (v4.2) Non-Word Reading	5/5	Within normal limits
MLSE (v4.2) Sentence Repetition	5/5	Within normal limits
MLSE (v4.2) Written Instructions	5/6	Within normal limits
MLSE (v4.2) Picture Description	5/9	Spoken in first person and Poor grammar
MLSE (v4.2)<br>Motor Speech<br>Phonology<br>Semantics<br>Syntax<br>Total	 24/30<br>30/30<br>23/24<br>9/16<br>86/100	 Impaired<br>Within normal limits<br>Within normal limits<br>Impaired
**Nonverbal Skills**		
Doors & People – Copy of Shapes	12/12	Within normal limits
Rey Complex Figure Copy	34/36	Within normal limits
VOSP Object Decision	18/20	Pass
VOSP Number Location	10/10	Pass
VOSP Cube Analysis	9/10	Pass
**Attention**		
**WAIS-IV Digit Span**	10/16 (F = 7)<br>10/16 (B = 6)	Scaled Score = 10<br>Scaled Score = 12
**Executive Functions**		
Hayling<br>Initiation (time)<br>Inhibition (time)<br>Inhibition (score)	 11s<br>84s<br>4/15	 Profile Score = 5/7 (Mod. Average)<br>Profile Score = 4/8 (Low Average) *<br>Profile Score = 1/8 (Impaired) * * *
Brixton	16 errors	Profile Score = 6/10 (Average)
WAIS-IV Similarities	22/36	Scaled Score = 8 (borderline)
Mini SEA – Faux Pas Stories	17/30	Faux Pas Subscore = 9.75/15 * *
Mini SEA – Facial Emotion Recognition<br>Happiness<br>Fear<br>Disgust<br>Anger<br>Surprise<br>Sadness<br>Neutral<br>Total Subscore	5/5<br>2/5<br>4/5<br>2/5<br>5/5<br>1/5<br>5/5	Within normal limits<br>Impaired * *<br>Within normal limits<br>Impaired * *<br>Within normal limits<br>Impaired * * *<br>Within normal limits (multiple false positives)<br>Emotion Subscore = 10.29/15 * *
**Mini-SEA Total Score**	**--**	**Total Score = 20.04 * * ***
**Other**		
HADS Anxiety Scale	0/21	Within 'normal' range
HADS Depression Scale	0/21	Within 'normal' range
