# Supplementary material for: Multisystem pathology in McLeod syndrome
Source: Neuropathology. Author manuscript; Available in PMC 2026 Oct 16. (PMC7619572; doi:10.1111/neup.12935)
Supplement: Supplementary file 3 [file EMS220163-supplement-Supporting_Information.pdf]

SUPPORTING INFORMATION

Additional supporting information may be found in the online version of this article at the publisher's website: http://onlinelibrary.wiley.com/doi/10.1111/neup.12935/suppinfo.

Table S1. Neuropsychological score sheet.
Table S2. Results of nerve conduction studies (NCS) and electromyography (EMG).
Video S1. Video of clinical examination illustrating choreiform movements in arms and reduced body mass index due to inadequate oral intake because of the oromandibular dyskinesias (not visible).
Video S2. Video of clinical examination illustrating reduced deep tendon reflexes.
Video S3. Video of clinical examination illustrating wide-based gait and reduced body mass index due to inadequate oral intake because of the oromandibular dyskinesias. A gastrostomy feeding tube was inserted soon after the video was taken.

© 2023 The Authors. *Neuropathology* published by John Wiley & Sons Australia, Ltd on behalf of Japanese Society of Neuropathology.

14401789, 2024, 2, Downloaded from https://onlinelibrary.wiley.com/doi/10.1111/neup.12935 by Test, Wiley Online Library on [01/04/2024]. See the Terms and Conditions (https://onlinelibrary.wiley.com/terms-and-conditions) on Wiley Online Library for rules of use; OA articles are governed by the applicable Creative Commons License
